# Measuring Quality of Life in Type 2 Diabetic Patients at the Al-Wazarat Healthcare Center in Riyadh

**DOI:** 10.7759/cureus.6474

**Published:** 2019-12-27

**Authors:** Abdullah S Albader, Saleh Albahlei, Mishary Almishary

**Affiliations:** 1 Family Medicine, Al-Wazarat Health Care Center, Riyadh, SAU

**Keywords:** determinants, qol, quantifying, diabetes, diabetic

## Abstract

Background

The prevalence of type 2 diabetes in the Saudi population is increasing at an alarming level. Diabetes is characterized by a considerable health and economic burden on the population and affected individuals.

Objectives

This study aims to assess the level of quality of life in type 2 diabetic patients and to investigate the determinant of quality of life in a primary health care setting.

Methods

The study used a cross-sectional design to investigate the quality of life among type 2 diabetic patients at the Al-Wazarat Health Care Center (WHC) in Riyadh, Saudi Arabia. The study used the Arabic version of the quality of life 36-items short-form questionnaire (SF-36).

Results

The study included 482 completed questionnaires out of the 525 distributed. The response rate is 91.8%. The average age of the patients is 56.3 ± 7.8 years. The self-reported average body mass index (BMI) is 31.6 ± 6.6 kg/m2. The duration of diabetes since diagnosis is 9.7± 3.1 years. The most common comorbidity was hypertension 75.9% (366/482). The multivariate regression analysis provided models that explained the role of certain variables in determining the quality of life in type 2 diabetic patients significantly. The most striking results are explaining the factors affecting physical functioning by 41% (R2=0.41) and mental health by 34% (R2=0.34).

Conclusion

This study can influence the practices of medical practice and promotion in WHC specifically and Riyadh city more generally. The improvement and preservation of HRQoL in diabetic patients required an understanding of the factors that can influence it. The gender disparity is an area that needs further investigation. Changes in the delivery of healthcare in diabetes clinics to account for these factors may provide better results.

## Introduction

Diabetes is a growing problem globally with a great impact on societies and individuals at the health and economic levels [1-2]. Saudi Arabia is one of the most affected countries, with 21.7% of the estimated prevalence of type 2 diabetes mellitus [3]. Lifestyle restrictions and diabetes complications can have a considerable impact on the patient’s quality of life (QoL) [4]. Due to the impact on the affected individuals, measuring these individuals’ health-related quality of life (HRQoL) in Type 2 DM is important for a wide range of reasons, from diet and lifestyle restrictions to controlling symptoms, comorbidities, and treatments administration. All of these factors may lead to negatively affecting QoL. Additionally, it is one of the healthcare service objectives especially per diabetes guidelines to improve the patients’ HRQoL [5-6].

The measuring tool was chosen after careful consideration of the literature. A review of the tools used to measure HRQoL in diabetic patients did not lead to the same results [7]. The authors concluded “No single measure can suit every purpose or application but when measures are selected inappropriately and data misinterpreted, any conclusions drawn are fundamentally flawed. If we value QoL as a therapeutic goal, we must ensure that the instruments we use are both valid and reliable.” [7]. Two systematic reviews showed that the most frequently used tools in HRQoL are the World Health Organization Quality of Life (WHOQOL) and SF-36 [7-8]. After consideration, we considered SF-36 as the tool for our study because of the readily validated Arabic version, the ease of administration, and the short time required to finish it [9]. The most comprehensive sources we found are two systematic reviews about the studies associated with sociodemographic and disease-specific variables [8-14]. Verna et al., in a 2017 study confirmed, using SF-36, several factors that influenced the HRQoL in 537 patients [15]. The investigation showed a negative association between depression symptoms, duration of diabetes, glycated hemoglobin (HbA1c) > 7%, and micro- macrovascular complications with HRQoL. The study also reported that being male and physically active associate positively with HRQoL [15].

## Materials and methods

Study area

A cross-sectional study was conducted at the Al-Wazarat Health Centre (WHC). Type 2 diabetic patients with files and records for at least one visit and 18 years or older were included in the study. Type 2 diabetic patients who do not have records at the WHC and who are from outside Riyadh were excluded from the study.

The research tool

The research tool is the Arabic version of the QoL SF-36 by Rand corporation [9]. The SF-36 is a self-report, 36-item survey measuring the health-related quality-of-life. Thirty-five items are used to construct eight scales. An additional item measures health transition. The questionnaire included sociodemographic questions and medical history questions. The sociodemographic questions included age, gender, weight (self-reported), marital status, height (self-reported), education, and employment status.

Statistical analysis

The statistical analysis in this study consisted of two parts. The first part consists of the non-theoretical descriptive statistics and the second part consists of hypothesis testing. The hypothesis testing based on the assumption that the score of each domain in the questionnaire is a continuous, normally distributed variable. Each of the scores will be considered a dependent variable and will be regressed using ordinary least squares (OLS) regression against strings of independent variables. The 'best-fitting' model then will be selected using the information criterion (IC). There are two information criteria widely used for model selection. The first is Akieke's Information Criterion (AIC) and Bayesian Information Criterion (BIC). The idea of IC is choosing the model that gives the best explanation (R2) with the least complexity. Introducing variables to the regression model always improves the explanation of the model, but it also increases the complexity of the model with the newly added variables. AIC and BIC try to keep a trade-off between the amount of improvement and complexity added by the new variables. The 'best' model is the model that minimizes the IC. We will use AIC for our analysis, as it is more theoretically established.

## Results

Table 1 describes the sample. The final sample size consisted of 482 completed questionnaires out of the 525 distributed. This accounts for a 91.8% response rate. The average age of the patients questioned was 56.3 ± 7.8 years. The majority of the respondents were men (63.1% (302/482)). The participants were mostly married (80.7% (389/482)). The education level of the participants was concentrated in secondary (43.8% (211/482)) or elementary school ((149/482)). Unemployment represented the largest portion of 60.4% (291/482). The income category most reported was household income 3,001 - 6,000 SAR (36.9% (178/482)). The self-reported average BMI was 31.6 ± 6.6 kg/m^2^. The duration of diabetes since diagnosis was 9.7 ± 3.1 years.

**Table 1:** Sociodemographic and participant characteristics DM: diabetes mellitus; BMI: body mass index

Characteristic	Mean ±SD	Frequency	Percentage
Age (years)	56.3 ± 7.8		
Gender			
Male		302	63.1
Female		180	36.9
Total		482	100.0
Marital Status			
Unmarried (Single, Divorced, Widowed)		93	19.3
Married		389	80.7
Total		482	100.0
BMI**	31.6 ± 6.2		
Education			
Illiterate/no formal education		86	17.8
Elementary		149	30.9
Secondary		211	43.8
College or higher		36	7.5
Total		482	100.0
Employment			
Employed		191	39.6
Unemployed or retired		291	60.4
Total		482	100.0
Income (SAR)			
<3,000		104	21.6
3,001 – 6,000		178	36.9
6,001 – 9,000		96	19.9
9,001 – 12,000		63	13.1
> 12,000		41	8.5
Total		482	100.0
Duration of DM (years)	9.7 ± 3.1		

The patients' history of diabetes-related morbidities recording the patient’s intake of insulin was also recorded. Retinopathy showed the highest microvascular complication (21.0% (101/482)). Cardiovascular prevalence was high in the sample with 28.4% (137/482) reported cardiovascular diseases. Hypertension is the most common diabetic-related comorbidity (366 (75.9%)). Insulin intake was reported by 18.9% (91/482). The patients' history of diabetes-related morbidities is summarized in Table 2.

**Table 2:** Patients' history of diabetes-related morbidities * CVD: cardiovascular disease

	Description	Frequency (%)
Microvascular complications	Retinopathy	101 (21.0)
	Neuropathy	59 (12.2)
	Nephropathy	16 (3.3)
Macrovascular complications and comorbidities	CVD*	137 (28.4)
	Hypertension	366 (75.9)
	Hyperlipidemia	194 (40.2)
Insulin intake	Yes	91 (18.9)

Central tendency measures and reliability of the SF-36 scales are presented in Table 3. Concerning reliability, all scales meet the recommended >0.70 internal consistency criterion. The eight subscale scores range from 46.2 for GH to 82.4 for SF.

**Table 3:** Central tendency measures and reliability of the SF-36 subscales

Scales	Mean ± SD	95% CI	Median	Reliability
Physical Functioning	61.6 ± (26.8)	59.2 – 64.0	68.0	0.89
Role Physical	59.8 ± (48.3)	55.5 – 64.1	75.0	0.93
Bodily Pain	67.2 ± (28.5)	64.7 – 69.7	82.0	0.91
General Health	46.2 ± (26.7)	43.8 – 48.6	48.0	0.81
Vitality	61.3 (32.8)	58.4 – 64.2	65.0	0.84
Social Functioning	82.4 (26.9)	80.0 – 84.0	90.5	0.96
Role Emotional	73.0 (39.6)	69.5 – 76.5	100.0	0.91
Mental Health	54.4 (30.1)	51.7 – 57.1	60.5	0.82

Regression analysis

HRQoL determinants are investigated using multivariate regression. All sociodemographic and diabetes-related disease variables are included in the full model. The best model is selected using AIC as indicated previously in the methodology section. Table 4 shows the results of the regression analysis. The models for the SF-36 (Table 4) showed that sex (female) had a negative effect across all sections in the SF-36. Other sociodemographic factors were significant predictors for certain SF-36 subscale scores, aging was associated with lower physical functioning (PF), bodily pain (BP), and role emotional (RE) scores (P < 0.01), being married with higher general health (GH), vitality (VT), and mental health (MH) (P < 0.05), higher education with less VT (P < 0.05) and being employed with worse GH (P < 0.01) and higher BP and MH (P < 0.05). The association with diabetes-specific factors, microvascular complications, and diabetes duration were the most influential on HRQoL, each factor associates negatively and statistically significantly with five and four SF-36 subscales, respectively. The PF construct shows the highest influence of the factors investigated. This is mainly reflected by the variance explained, that is, 41%. The rest of the subscales, the models explained the portions of variance ranging between 9% and 34%.

**Table 4:** Multivariate linear regression analysis for the SF-36 scales BMI: body mass index; PF: physical functioning; RP: role physical; BP: bodily pain; GH: general health; VT: vitality; SF: social functioning; RE: role emotional; MH: mental health

	PF	RP	BP	GH	VT	SF	RE	MH
Constant	140.6	86.2	101.3	24.8	64.9	98.4	122.4	46.9
	(p <0.001)	(p <0.001)	(p <0.001)	(p =0.024)	(p <0.001)	(p <0.001)	(p <0.001)	(p <0.001)
Age (per year)	-1.2		-0.6				-1.1	
	(p <0.001)		(p =0.028)				(p =0.018)	
Female	-21.3	-24.6	-18.2	-9.0	-14.7	-23.9	-31.5	-16.6
	(p <0.001)	(p <0.001)	(p <0.001)	(p <0.001)	(p <0.001)	(p <0.001)	(p <0.001)	(p <0.001)
Married				1.5	11.3			1.6
				(p <0.001)	(p <0.001)			(p =0.048)
College or higher					3.8			
					(p <0.001)			
Employed			3.8	-11.3				6.6
BMI per kg/m2	-1.8		(p =0.031)	(p <0.001)				(p <0.001)
	(p <0.001)							
Microvascular	-20.3			-19.1		-17.4	-13.5	-14.2
Complications	(p <0.001)			(p <0.001)		(p <0.001)	(p =0.016)	(p <0.001)
Macrovascular	-18.5			-5.7	-9.1			
Complications	(p <0.001)			(p =0.036)	(p =0.008)			
Hypertension				-8.2				-4.1
				(p =0.011)				(p=0.041)
Hyperlipidemia								-10.8
								(p =0.006)
Duration of diabetes (per	-6.5		-0.9	-0.4	-0.8			
year)	(p<0.014)		(p <0.001)	(p =0.032)	(p =0.009)			
Insulin intake		-8.2		-13.4	-8.1			-6.9
		(p <0.013)		(p <0.001)	(p <0.001)			(p <0.026)

## Discussion

In this work, we investigated HRQoL and the factors that may influence HRQoL in diabetic patients in Saudi Arabia in primary care settings.

Sociodemographic and diabetes-specific variables were included in the investigation tool. Our investigation confirmed the importance of several diabetes-specific variables as per the literature. Our investigation confirmed obesity (per unit increase in BMI) [14-22], comorbidities such as hypertension [21,23-24], and dyslipidemia [21,24-25], and micro- and macrovascular complications [14, 21,24]. The sociodemographic variables that influenced HRQoL are age [14,21,26], gender [14,21,26], marital status [14,21,26], and higher education [14,21,26]. These variables showed a very interesting influence on HRQoL, especially the notable differences between males and females.

The multivariate regression analyses indicate that while sociodemographic variables can be important, specifically gender, diabetes-related variables are more important predictors of HRQoL. Females are overall influencing the subscales as an exception. Microvascular complications, disease duration, and comorbidities were the most profound predictors of a negative QoL. The combination of these variables seems to explain a large part of the variability in most subscales. Only RP shows resistance to the factors investigated. Only 9% of the variability in this subscale is explained. In contrast, because of the relationships observed in this study and previous studies, it is plausible to conclude that trying to avoid obesity, delaying the development of complications, hypertension, hyperlipidemia, and other non-diabetic comorbid conditions will enhance HRQoL. Such enhancement can lead to improved life expectancy as well. Unfortunately, the current practice overlooks the impact of the treatment and the complications' effect on the HRQoL in the prevention protocols. Such a gap between implemented treatments and prevention policies and the patient’s HRQoL opens the door for questioning the extent of treatment satisfaction among patients [27]. The results of this study conform to the findings in several previous studies. The sociodemographic variables are major influences, specifically being a female patient on the HRQoL. This result was profound in our study. The high prevalence of some complications and comorbidities showed that diabetic-specific comorbidities are major influences.

Limitations

The study has several limitations. The cross-sectional design of the study only allows for the casual association, not causality. The single-center setting means that the results should be generalized cautiously. The low response rate among the older patients cast some doubts over some of the results due to the lack of representation of these older age groups. Finally, the study overlooked assessing the patients psychologically and such factors can be very detrimental in the patient’s perception of HRQoL.

## Conclusions

This study can be utilized in the practices of healthcare and medicine promotion in WHC specifically and in primary care settings in Riyadh city more generally. Improving HRQoL in diabetic patients requires an understanding of the factors that can influence it. Changes in the delivery of healthcare to be more personalized in diabetes to account for these factors may provide better results. We recommend (a) continuous measuring of HRQoL in diabetes to keep understanding the determinants of HRQoL in T2DM, (b) changes in healthcare provided to T2DM patients for more personalized medical care that can account for the disparities between individuals, and (c) educational and psychological support especially for female patients.
